# Convolutional Neural Network for Fully Automated Cerebellar Volumetry in Children in Comparison to Manual Segmentation and Developmental Trajectory of Cerebellar Volumes

**DOI:** 10.1007/s12311-023-01609-2

**Published:** 2023-10-13

**Authors:** Daria Juliane Sobootian, Paul Bronzlik, Loukia M. Spineli, Lena Sophie Becker, Hinrich Boy Winther, Eva Bueltmann

**Affiliations:** 1 Institute of Diagnostic and Interventional Neuroradiology, Hannover Medical School, Hannover, Germany; 2 Midwifery Research and Education Unit, Hannover Medical School, Hannover, Germany; 3 Institute of Diagnostic and Interventional Radiology, Hannover Medical School, Hannover, Germany

**Keywords:** Cerebellum, Magnetic resonance imaging, Automated segmentation, Neural network, Pediatrics

## Abstract

The purpose of this study was to develop a fully automated and reliable volumetry of the cerebellum of children during infancy and childhood using deep learning algorithms in comparison to manual segmentation. In addition, the clinical usefulness of measuring the cerebellar volume is shown. One hundred patients (0 to 16.3 years old) without infratentorial signal abnormalities on conventional MRI were retrospectively selected from our pool of pediatric MRI examinations. Based on a routinely acquired 3D T1-weighted magnetization prepared rapid gradient echo (MPRAGE) sequence, the cerebella were manually segmented using ITK-SNAP. The data set of all 100 cases was divided into four splits (four-fold cross-validation) to train the network (NN) to delineate the boundaries of the cerebellum. First, the accuracy of the newly created neural network was compared with the manual segmentation. Secondly, age-related volume changes were investigated. Our trained NN achieved an excellent Spearman correlation coefficient of 0.99, a Dice Coefficient of 95.0 ± 2.1%, and an intersection over union (IoU) of 90.6 ± 3.8%. Cerebellar volume increased continuously with age, showing an exponentially rapid growth within the first year of life. Using a convolutional neural network, it was possible to achieve reliable, fully automated cerebellar volume measurements in childhood and infancy, even when based on a relatively small cohort. In this preliminary study, age-dependent cerebellar volume changes could be acquired.

**Supplementary Information:**

The online version contains supplementary material available at 10.1007/s12311-023-01609-2.

## Introduction

The cerebellum is well known to play an essential role in motor function. Because of its broad and complex involvement in cognition, emotional control, and social abilities, scientific interest in the cerebellum has grown, with multiple studies performed over the last decades [1–4]. Especially the developing cerebellum appears to be a challenging research subject, considering the disturbance of its development may result in neurodevelopmental disorders and cognitive and neuromotor deficits [5, 6].

The human brain undergoes rapid maturation, especially in the first few years of life. The cerebellum is no exception. Commencing in utero in the early first trimester, cerebellar development requires roughly until the second postnatal year to achieve full circuit maturity [7]. The growth rate is awe-inspiring. In comparison to other brain structures, the cerebellum has the highest growth rate, more than doubling its size in the first 90 days after birth [8]. It continues to grow rapidly and thus reaches a total increase of 240% at the end of the first year of life, while the entire brain undergoes a percentage volume change of only 101% [9]. In the second year of life, growth flattens out, resulting in a total growth increase of 15% [9]. With the knowledge of the cerebellar volume changes during the first 2 years of life, this time is presumed to be highly vulnerable, highlighting the importance of pathology detection at an early stage in their development.

Due to its high spatial resolution and excellent image contrast without radiation exposure, MRI is the method of choice for diagnosing many clinical conditions in children. The cerebral development can be made visible [9], including physiological as well as pathological changes. Routinely, image analysis is performed qualitatively and is, therefore, highly dependent on the investigator. Quantitative analysis techniques such as T1 and T2 relaxation times, diffusion anisotropy, and magnetization transfer that support the interpretation of image data are desirable to detect discrete pathologies as early as possible and investigator-independent. Determining the cerebellar volume is an essential criterion in addition to describing its signal intensities and structure when assessing the cerebellum. Several studies have already addressed the relationship between cerebellar volume in different patient groups and various diseases or even certain environmental factors, such as ADHD [10–14]. Furthermore, the relations between the total volume of the brain and the volume of specific subdivisions, e.g., the cerebellum, in children with specific diseases have been repeatedly researched using different segmentation methods [15, 16]. These observations require precise volumetric measurements, which are very time-consuming, require a profound knowledge of cerebellar anatomy, and are also highly error-prone [17, 18]. Numerous methods for automated segmentation of medical imaging data have been introduced to address this challenging task over the years [17, 19, 20]. Multi-atlas label fusion techniques have emerged in the last decade. Even further development in that area was achieved with the introduction of deep learning techniques, such as the convolutional neural network presented in this study. Several studies applied these algorithms to calculate cerebellar volume [21, 22]. However, these studies were primarily performed in adults, given that segmenting cerebral structures in children, especially in newborns, is challenging due to their small size and reduced contrasts in imaging [23]. Furthermore, imaging data of inconspicuous healthy children are often not available.

The aim of the study was firstly to develop a fully automated and reliable volumetry of the cerebellum of children during infancy and childhood based on 3D T1-weighted images and deep learning algorithms and secondly to demonstrate the clinical significance of this investigator-independent, quantitative measurement.

## Materials and Methods

The local institutional review board approved this retrospective study. All parents provided written informed consent to a scientific evaluation. From our database of pediatric MRI examinations between 2014 and 2020, one hundred investigations scanned at 1.5T (Magnetom Aera, Siemens, Erlangen, Germany) using a 20-channel head coil were retrospectively selected, showing morphologically inconspicuous images without signal abnormalities as assessed by an experienced neuroradiologist (E.B., more than 15 years of experience). Brain MR imaging was performed for various clinical indications, including vomiting, headache, primary onset of epileptic seizures, tumor exclusion, or mild traumatic brain injury. Exclusion criteria were any signs of an infratentorial pathology and motion artifacts. Patient ages ranged between 0 and 16.3 years old (47 females, 53 males), with a mean age of 4 years and 50% younger than 24 months. Our routine MR protocol included a 3D T1-weighted magnetization prepared rapid gradient echo (MPRAGE) sequence with the following parameters: 1 mm continuous slice thickness, matrix 256 × 246, TR 2200 ms, TE 2.67 ms, and flip angle 8°. The acquired data sets were converted into NIFTI files to use the open-source Segmentation program ITK-SNAP for postprocessing [24] (version 3.8.).

### Segmentation Process

**Fig. 1:**
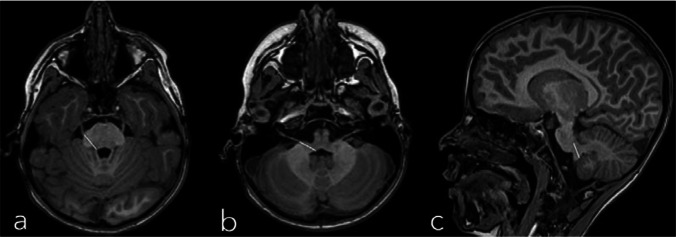
**a**–**c** Standardized manual approach for delineating the brainstem from the cerebellum

Manual segmentation was performed mainly in the axial plane. To have a standardized approach for delineating the brainstem from the cerebellum and to ensure that the cerebellar peduncles were correctly included, we used the method presented by Weier et al. [25]. Step one was to determine the image plane on which the cisternal part of the trigeminal nerve was visible. The same was done for the vestibulocochlear nerve. We then drew a line from each trigeminal nerve to the upper cerebellar peduncle (Fig. 1a) and in the extension of each vestibulocochlear nerve to the fourth ventricle (Fig. 1b), defining the rostral border of the cerebellum. Finally, the interpolation tool was used between these two layers to determine the rostral segmentation border in between (Fig. 1c).

Based on this delineation, the primary investigator, a specially trained graduate student, started each systematic manual segmentation of all 100 cases to teach the network the proper boundaries of the cerebellum. All 100 data sets were then manually segmented.

We utilized a four-fold cross-validation, dividing the 100 cases into four different splits. Each split contains 75 training and 25 testing cases. Each case was included once in the testing split, meaning no child was studied twice. The data selection for the four different splits was balanced by age. This was achieved by sorting the data sets by age and selecting each fourth entry using an offset of zero to three for the respective split.

Four separate neural networks were trained on each training split of the four-fold cross-validation. Two random exams of the respective training split were chosen to calculate performance metrics during training. The best-performing model was selected as the final model for the respective split based on the training metrics.

### Network Topology and Training

We used a 3D U-Net architecture derived from Çiçek et al. [26], as previously published [27]. It was implemented in TensorFlow [28]. The input layer has been expanded to 256×256×256 neurons. Each downsample block consists of a batch normalization followed by two 3×3 convolutions with a 2×3 striding with zero padding. Each upsample block consists of a resampling layer, resizing the input to the same resolution as the corresponding downsample block using linear interpolation. This is followed by two 3×3 convolutions with zero padding. The output layer is the same size as the input layer. As suggested by He et al. [29], all activation functions have been converted from rectified linear units (ReLU) to parametric rectified linear units (PReLU). The neural network was trained by minimizing the weak binary cross-entropy as an objective function. We started with a learning rate of 1e-3 and gradually lowered it to 1e-6 during the training. Each epoch consisted of 200 virtual samples (steps) drawn dynamically from the image augmentation with a batch size of 2. We used linear transformations, such as rotation, shearing, and resizing, as well as non-linear transformations, such as local displacement for image augmentation.

The final model of each split was selected based on the performance metrics during training. The first model was trained for 113, the second for 159, the third for 386, and the fourth model for 143 epochs. We chose Adaptive Moment Estimation (Adam) as the stochastic optimization method. The training was performed on an eight-core Intel Xeon CPUE5-2637 v4 @ 3.50 GHz system with 128 GB of RAM and two NVIDIA Tesla P40 GPUs with a total of 24 GB of video memory each and ran for 7 days.

Each of the resulting four networks was then used to perform an image segmentation of the 25 test cases for the respective split. No preprocessing, such as denoising or brain extraction, was performed.

To ensure reproducibility, 25 selected data sets (the testing split of one split of the four-fold cross-validation) were manually segmented a second time by the primary investigator. Here, too, a balanced age distribution was given. Furthermore, the same 25 data sets were assigned to an experienced neuroradiologist (E.B.) for manual segmentation to obtain inter-rater reliability.

### Segmentation Quality Metrics

In this work, we provided metrics, such as the Dice coefficient, also known as Sørensen–Dice coefficient, and intersection over union (IoU), also known as the Jaccard index.

### Volumetric Measurements

Based on the ground truth, the cerebellar volumes were calculated to obtain healthy children’s age-dependent cerebellar volume growth. Unexpectedly large and small cerebellar volumes existed. We, therefore, examined their medical history in more detail and looked for reasons for these deviations. Based on their medical history, the outliers belonged to children with various neurodevelopmental disorders and concomitant micro- or macrocephaly, so we removed these 13 cases to describe normal age-related cerebellar development.

### Statistical Analysis

We used violin plots with incorporated box plots to illustrate the volume distribution (in ml) for each sex and age group (age categorized into distinct, relatively balanced groups). We performed two multivariable ordinary least squares regression analyses: (i) regressing the volume against the age in years (in the logarithmic scale), sex, and head size (model 1), and (ii) removing head size from the model (model 2). We compared these two models regarding the adjusted *R*^2^ and the Akaike information criterion (AIC). The model with the largest adjusted *R*^2^ and a smaller AIC by 2 points was preferred. For the selected model, we performed regression diagnostics to inspect the appropriateness of the model assumptions. We reported the regression coefficients (measured in mean difference) and the 95% confidence interval: a confidence interval that excludes zero (the null value) coincides with a *p*-value less than 5% and indicates statistical significance. We created the scatterplot with the fitted line for each sex separately, using different point shapes for the head size. All analyses and visualizations were implemented using the statistical software R (version 4.2.1) [30]. We used the R-package *AICcmodavg* for the AIC results and the R-package *ggplot2* for the visualizations mentioned above [31, 32].

### Comparison to Prior Work

We compared our results with the predictions of ACAPULCO and FreeSurfer. We use the CPU version 0.3.0 of ACAPULCO pediatric as a docker container provided by the original authors: registry.gitlab.com/shuohan/acapulco:0.3.0 (Image ID: a9a95b207bf8). FreeSurfer was utilized in version 7.4.1 as a docker container provided by the FreeSurfer project: freesurfer/freesurfer:7.4.1 (Image ID: 2ce88773a7f6).

## Results

### Automated Segmentation of the Cerebellum

**Fig. 2:**
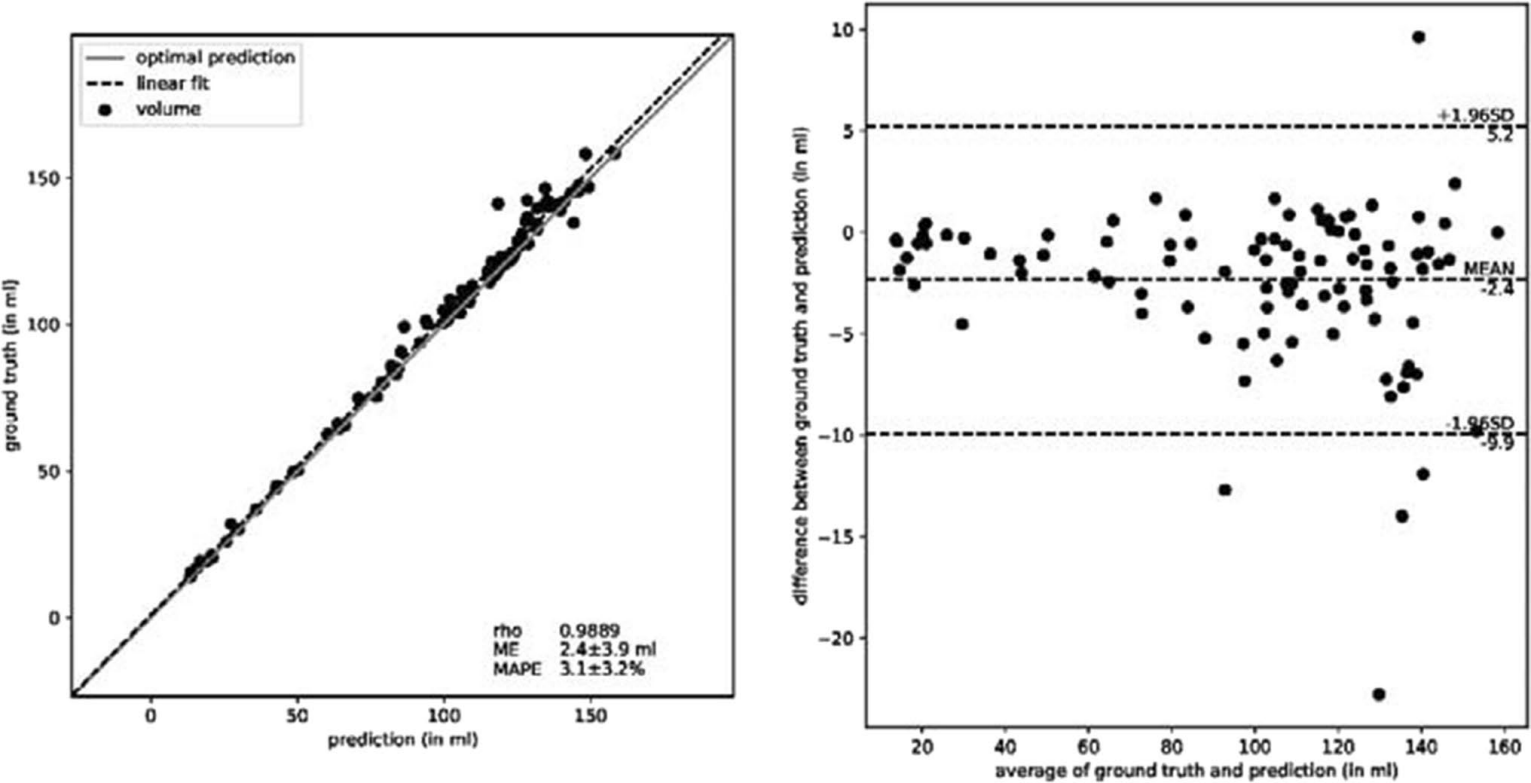
Display of congruence between the ground truth volume, determined by the manual segmentation, and the volume prediction by the neural network

As shown in Table 1, our trained neural network achieved an overall Spearman correlation coefficient of 0.99, a Dice Coefficient of 95.0 ± 2.1%, and an IoU of 90.6 ± 3.8% with the ground truth as shown in Fig. 2. The mean absolute difference amounts to 2.4 ± 3.9 ml. The mean absolute percentage error (MAPE) is 3.1 ± 3.2%.

**Table 1:**
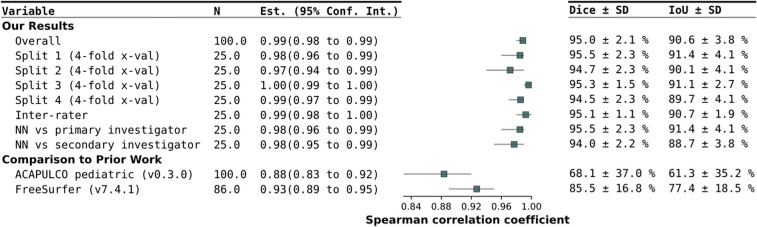
The Spearman rank-order correlation coefficient (based on the predicted and ground truth cerebellar volume), confidence intervals, Dice Coefficient, and IoU metrics have been calculated for the trained neural network (including the different splits of the four-fold cross-validation), inter-rater-correlation, and comparison with the primary and secondary investigator as well as for ACAPULCO and FreeSurfer. The metrics of ACAPULCO and FreeSurfer are in regard to the manual ground truth segmentation

**Fig. 3:**
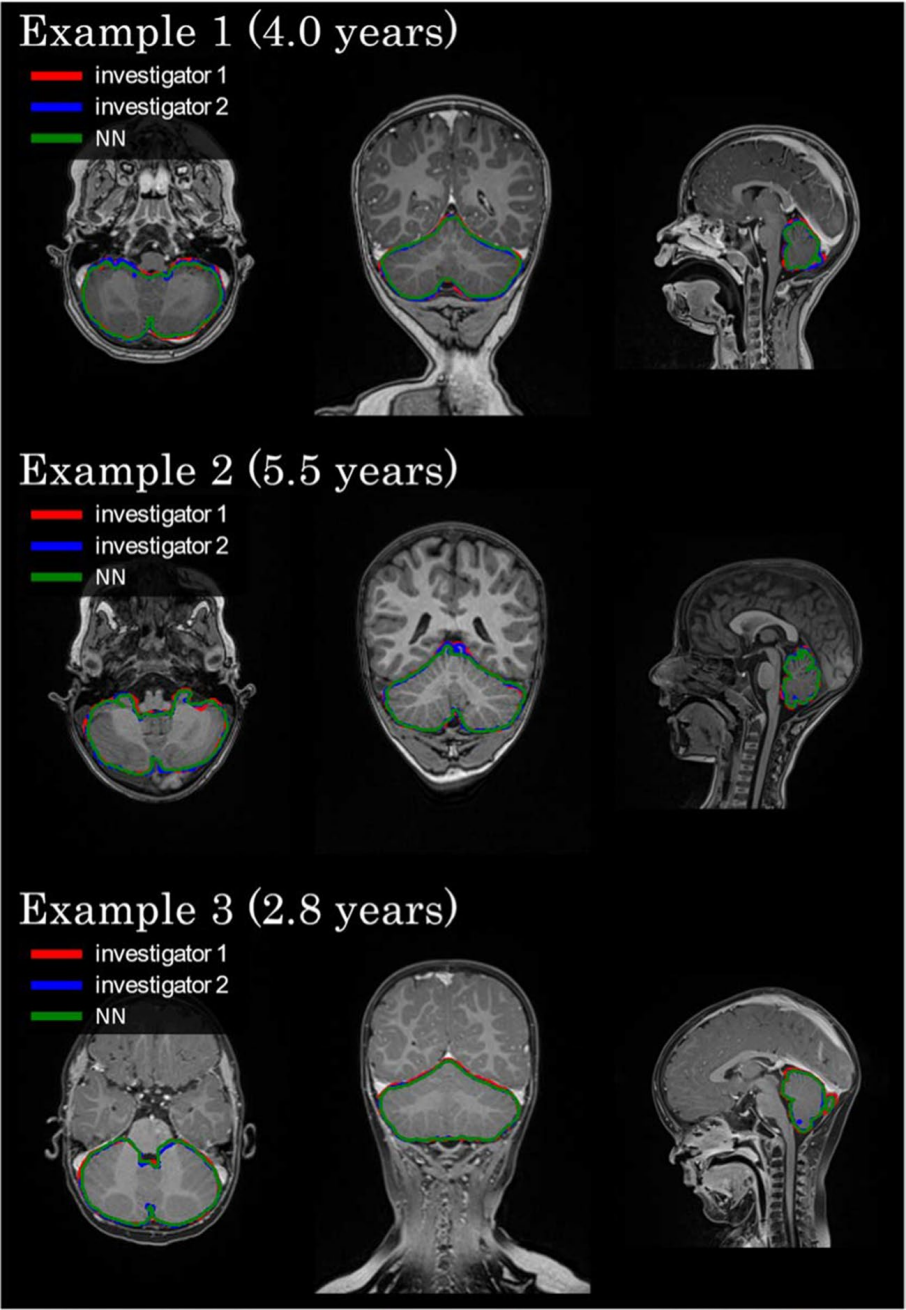
Three examples of segmentations depicting the manual delineation from investigator one and investigator two and the neural network’s prediction

The inter-rater correlation can also be derived from Table 1. We achieved a Spearman correlation coefficient of 0.99, a Dice Coefficient of 95.1 ± 1.0%, and an IoU of 90.7 ± 1.9% from the 25 subjects examined by both our investigators. In addition, the accuracy of the newly created neural network was compared with the manual segmentation of our primary investigator and secondary investigator based on these 25 subjects. It showed a Spearman correlation coefficient of 0.98, a Dice Coefficient of 95.5 ± 2.3%, and an IoU of 91.4 ± 4.1% for the neural network in comparison to the primary investigator and a Dice Coefficient of 94.0 ± 2.2%, and an IoU of 88.7 ± 3.8% compared to the secondary investigator. These results are shown graphically in Fig. 2. Three examples of segmentations comparing the manual segmentation from investigator one and investigator two and the neural network's prediction are shown in Fig. 3.

### Comparison to Prior Work

We compared our results to the pediatric parcellation protocol of the ACAPULCO pipeline [33] provided by Han et al. (version 0.3.0 CPU). ACAPULCO achieved a Spearman correlation coefficient of 0.883, a Dice coefficient of 68.1 ± 37.0% (mean ± std), and an IoU of 61.3 ± 35.2% compared to the manual ground truth segmentation.

FreeSurfer reached a Spearman correlation coefficient of 0.93, a Dice Coefficient of 85.5 ± 16.8%, and an IoU of 77.4 ± 18.5% compared to the manual ground truth segmentation. A total of 86 cases were processed successfully. Of the 14 missing cases, 12 failed the Talairach check, one triggered a watershed error due to a failed brain region detection, and the skull stripping failed for one case. The 14 missing cases were omitted when calculating the IoU and volume metrics. Twelve of the 14 missing cases were under 1 year old.

### Age-Dependent Manually Segmented Volumetric Measurements

Table 2 describes the relationship between cerebellar volume and age, demonstrating continuous growth. It illustrates the exponentially rapid growth in the first year of life. For male children, the calculated mean total cerebellar volume is higher in each age group, except for infants aged 0 to 6 months.

**Table 2:** Mean cerebellar volume for the different age groups

Age groups	*n* (m:f)	Total cerebellar volume (ml)				
		Range	Mean	Mean male	Mean female	SD
Age-dependent mean cerebellar volume for all 100 patients						
0–6.0 months	23 (14:9)	13.90–74.81	35.97	34.71	37.94	19.80
6.1–12.0 months	11 (7:4)	64.63–126.72	93.84	99.32	84.25	17.28
12.1–24 months	16 (8:8)	74.20–128.48	106.67	111.78	101.56	14.39
2–4 years	20 (10:10)	82.83–158.30	121.23	126.46	116.01	16.05
4–6 years	10 (5:5)	111.18–146.39	130.72	138.34	123.11	11.95
6–16 years	20 (9:11)	79.93–158.11	132.66	137.39	128.79	17.77
Total	100					
Age-dependent cerebellar volume for normocephalic patients without outliers						
0–6.0 months	23 (14:9)	13.90–74.81	35.97	34.71	37.94	19.80
6.1–12.0 months	9 (5:4)	64.63–108.79	89.59	93.87	84.25	14.65
12.1–24 months	14 (8:6)	84.84–128.48	107.46	111.78	101.70	11.04
2–4 years	14 (6:8)	104.80–134.24	118.40	119.89	117.28	8.45
4–6 years	10 (5:5)	111.18–146.39	128.36	133.62	123.11	11.14
6–16 years	17 (8:9)	118.18–158.11	139.67	143.17	136.56	9.01
Total	87					

**Fig. 4:**
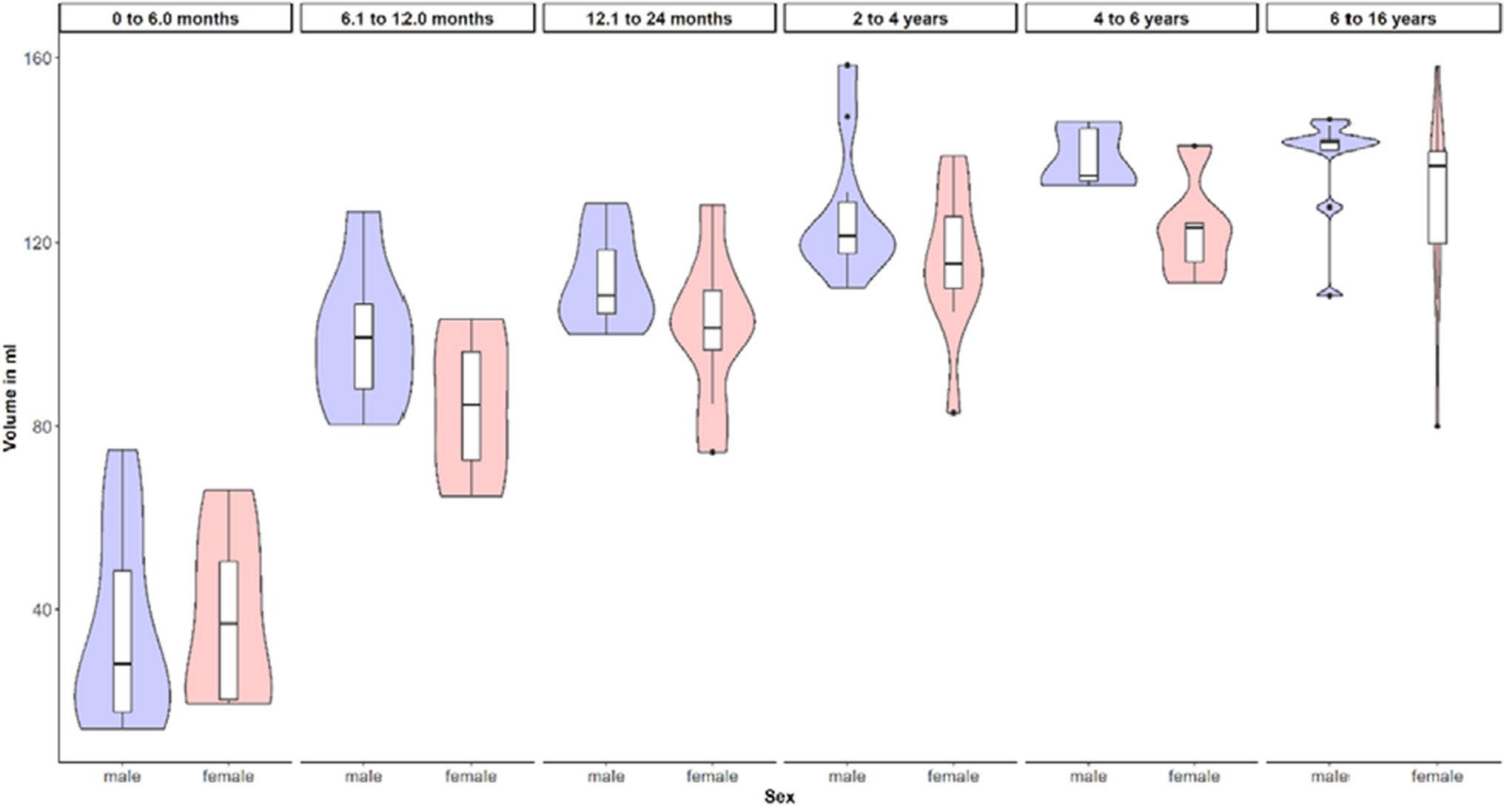
The panel of violin plots indicated a tendency for a larger volume in males than in females

The panel of violin plots also indicates a tendency for a larger volume in males than females. However, the violins with box plots overlap to some extent, and the pattern persists over the age groups (Fig. 4). The volume in both sexes increases exponentially with age, as expected.

### Regression Analysis

Model 1 was associated with a larger adjusted *R*^2^ and a substantially smaller AIC value (by 31.18 points); hence, it was considered in the present study (Table 3).

**Table 3:** Results from both regression models

Variable	Model 1^2^	Model 2^2^
Log age^1^	*19.67 (18.28, 21.06)*	*19.22 (17.62, 20.84)*
Sex (female)^1,3^	−5.19 (−10.54, 0.17)	−*7.57 (*−*13.84,* −*1.31)*
Head size (microcephaly)^1,3^	−*19.54 (*−*29.47,* −*9.60)*	–
Head size (macrocephaly)^1,3^	*29.46 (17.26, 41.65)*	–
Adjusted *R*^2^	90%	85%
AIC	808.45	839.63

*AIC* Akaike information criterion

^1^Results refer to mean difference and 95% confidence interval (in parenthesis)

^2^Results in italics refer to statistically significant results (i.e., zero value of no difference is not included in the 95% confidence interval)

^3^Parenthesis indicates the non-reference level of the corresponding variable: “female” for sex, “microcephaly” and “macrocephaly” for head size

The point estimate of the regression coefficients indicated a substantial association of the volume with all variables; however, there was a statistically significant association only with age and head size. Specifically, an older child had a larger volume on average than a younger child. Similarly, the volume increased by 29.46 ml on average (ranging from 17.26 to 41.65 ml) in a child with macrocephaly compared to normal head size. On the contrary, the volume decreased by 19.54 ml on average (ranging from 9.60 to 29.47 ml) in a child with microcephaly compared to normal head size. The results of the regression diagnostics can be found in the Supplementary material.

**Fig. 5:**
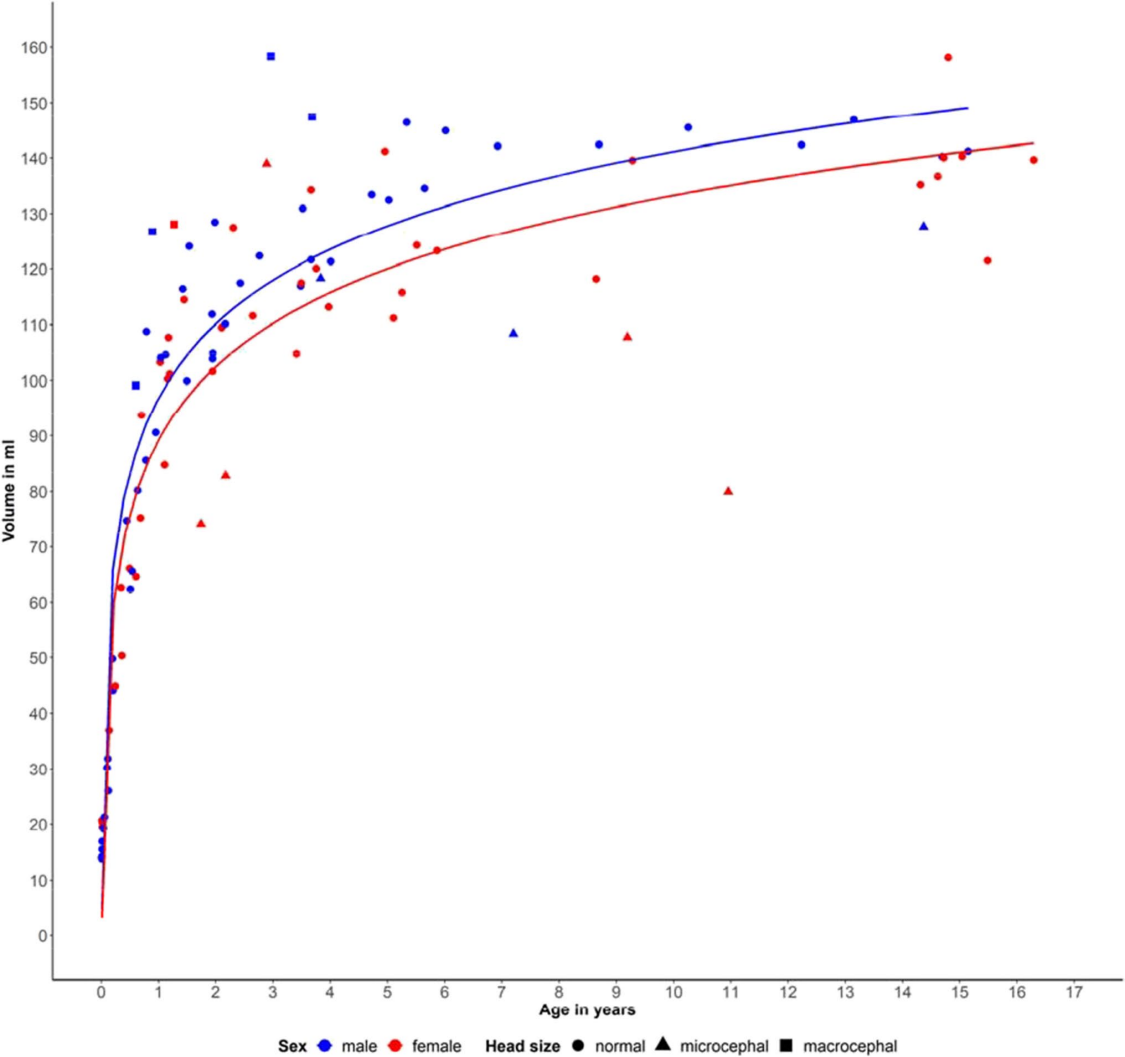
Age-dependent cerebellar volume growth of female and male children between 0 and 16.3 years of age based on the ground truth

Figure 5 depicts the individual cerebellar volume of each examined child depending on the age at the time of the MRI. The fitted regression lines by sex illustrate the exponential growth of the volume as the child grows, increasing quickly during the first 2 years of life. Consecutively, the growth rate decreases sharply, rendering the curve much flatter. A distinct age dependency of the cerebellar volume can be inferred: with increasing age in infancy, the cerebellar volume increases considerably. The lines are inseparable at the earliest age but start being separated after the first 6 months of life, with males tending to have a systematically larger volume than females. However, there are a few children with very low volume specifications below the expected curve that attract attention.

### Subgroup Analysis

The subgroup analysis, as depicted in Table 4, shows a statistically significantly worse prediction quality in the age group 0 – 6 months with a mean ± std dice coefficient of 0.93 ± 0.02, while the age groups 12 – 24 months and 2 – 4 years show a statistically significantly better prediction quality with a dice coefficient of 0.96 ± 0.02 and 0.96 ± 0.02 respectively. No statistically significant difference in quality is observed in the sex subgroups or regarding the different splits of the four-fold cross-validation.

**Table 4:**
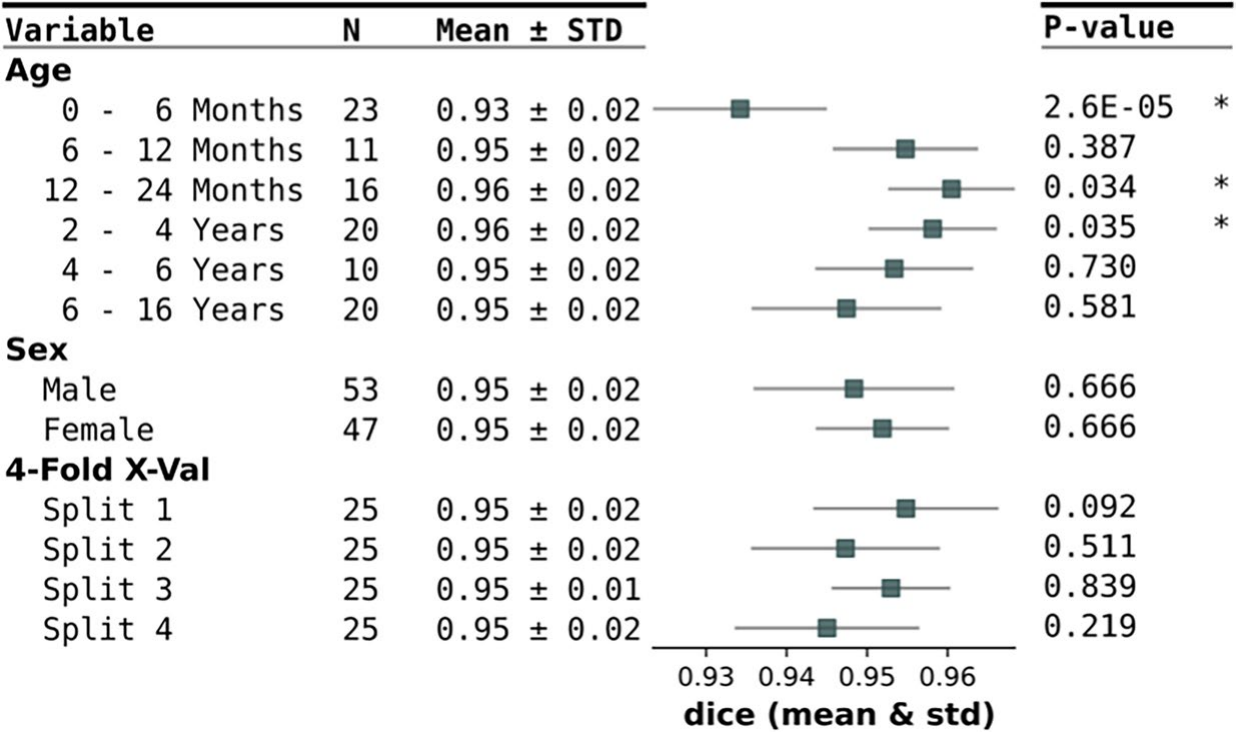
Subgroup analysis. Each subgroup was compared to the remaining data, e.g., split 1 was compared to split 2 – 4, respectively. The *p*-value was calculated using a Wilcoxon rank-sum test

## Discussion

One of the first approaches for a structured volume calculation was atlas-based, meaning the creation of different maps as a reference point for clinicians and investigators wanting to study the cerebellum [34]. While this segmentation tool for volume determination has been widely used for a long time, fully automated ones have only recently been explored [34–36]. A vast range of techniques and methods have already been applied, but up to date, the available automated methods for brain segmentation are mainly designed for adults instead of the pediatric population [37]. This is also reflected in our results when applying the FreeSurfer (version 7.4.1) to our data set, which yielded suboptimal results with a Dice coefficient of 85.5 ± 16.7% (mean ± std) and a Spearman correlation coefficient of 0.93. While some studies, such as Tiemeier et al. [11], included children, determining the normal cerebellar volume during childhood and adolescence, the parcellation was performed manually, and a distinctly higher age average was used compared to this study group with females being 13.7 years and males 13.9 years old. Narayanan et al. [38] introduced a probabilistic cerebellar atlas applicable to children’s data. However, the number of patients was limited to 18 subjects, and they included only children between 9 and 13 years.

An additional development of the standard atlas-based segmentation is the multi-atlas label fusion. Open data from several atlases were used to improve accuracy [38]. For the neonatal brain, this has been presented by Otsuka et al. [39]. While a huge number of atlases is required to achieve accurate results with a broad age coverage, deep learning techniques, especially deep convolutional neural networks, have been demonstrated to produce meaningful results even with comparatively small data sets [20]. However, training on a limited data set increases the risk of overfitting the model on the training data and may hurt generalizability.

Different imaging techniques, as well as different organ systems, have already used neural networks [17, 40]. The applicability of neural networks for parcellation of the cerebellum has been explored by Carass et al. [41] and Han et al. [33] for the age group of approximately 8 to 13 years. However, to our knowledge, no neural network is applicable to infant cerebellar volumes of any age from birth to adolescence. This is also reflected in our results where we compared our results to the pediatric parcellation protocol of the ACAPULCO pipeline (Han et al. [33] version 0.3.0 CPU), which achieved poor results with a Spearman correlation coefficient of 0.883 and a Dice coefficient of 68.1 ± 37.0% (mean ± std). The poor performance is most likely attributed to an overfit to the characteristics of the data set, i.e., ACAPULCO was trained on data from different MRI scanners (1.5T vs. 3T) with different protocol settings for the aforementioned age group.

The performance of our trained neural network is comparable—if not partly higher to similar studies with a Spearman correlation coefficient of 0.99 and a Dice coefficient of 95.0 [23, 38, 39, 42]. The results for the age-dependent subgroups were also excellent. The neonatal group (0–6 months old) performed statistically significantly worse compared to the other age groups, but even for these small babies with the lowest contrast due to incomplete myelination, the results were very good. Also, the calculated volumes of the cerebella correspond to the values determined so far in the literature. Wu et al. [43] showed cerebellar development in children using volumetric calculations in different age groups. In the younger age categories, mean volume was calculated at 77.5 cm^3^ for the 3- to 11-month-olds and 104.7 cm^3^ for the 1- to 1.9-year-olds, very similar to the volumes determined by the present study (75.44 ml for 3 to 11 months and 106.99 ml for 1- to 1.9-year-olds). Differences are seen in the age group of 4- to 5.9-year-olds, where they present a mean volume of 113.6 cm^3^ in comparison to a mean volume of 128.36 ml in the present study. Furthermore, the volume calculated in our study continues to increase (6 to 8.9 years 136.87 ml and 9 to 12 years 142.43 ml), while Wu et al. showed no significant increase. This may be due to different case numbers, given that the present study included only four children aged 6 to 8.9 years and three between 9 and 12 years. Another possible reason could be the consideration of different ethnicities since only Chinese children were included in the aforementioned research. In contrast, we investigated mainly Caucasian children. Kosar et al. [44] also determined the cerebellar volume in healthy children and adolescents between 6 and 17 years old by using stereological volume measurements and applying the point counting technique. They measured an average total cerebellar volume of 123.44 cm^3^; our result of 139.67 ml is considerably higher. The strongly varying number of cases in these age groups can explain this discrepancy. Kosar et al. studied 90 children aged between 6 and 17 years. In contrast, the number of patients in the present study belonging to this age group is significantly smaller with 17 children (8 males and 9 females). In addition, the sex ratio of the included subjects may have influenced the lower volume. Of the 90 examined children by Kosar et al., 50 were female, known to have a smaller cerebellar volume [11].

The present study has limitations. Especially for children <2 years, the number of included cases is relatively small, and acquiring an even larger patient collective would be desirable. However, MRI data from morphologically inconspicuous children are difficult to achieve with known risks of prerequisite sedation or intubation. Furthermore, we included pediatric patients with various clinical diseases in order to obtain normal age-related cerebellar measurements. This makes the group inhomogeneous and can be erroneous due to a possible underlying neurological disorder that was not evident at the time of inclusion. Furthermore, segmenting infantile cerebella within the first year of life can be difficult due to the low contrast between different brain regions, which might complicate the clear demarcation from surrounding structures. Additionally, we expect a certain degree of overfitting of the neural network, as this is a single-center study with a comparatively small and homogeneous data set.

Our goal was to generate a neural network that could reliably segment the cerebellar volume of children, which we could successfully prove despite the relatively small patient cohort. The calculated volumes corresponded well to those described in the literature. Furthermore, as shown by the outliers, quantitative analysis was superior to qualitative analysis because it could identify deviating volumes that were not visually recognizable.

In summary, convolutional neural networks are a feasible technique to achieve reliable cerebellar volume measurements in childhood and infancy, even when based on a relatively small cohort. The volumes generated from clinical MRI investigations correlated well with manually acquired data and were comparable to published literature. In the future, using the developed neural network, cerebellar volumes can be measured in an acceptable time frame as part of routine MR imaging. Furthermore, if the presented method is applied to a larger group of healthy children, normal age-dependent references can be acquired, reflecting normal age-dependent cerebellar volume changes. Volumetric measurements based on this method could then act as an investigator-independent, quantitative biomarker of cerebellar development and help to improve the radiological diagnosis.

### Supplementary Information

ESM 1 (DOCX 204 kb)

## Data Availability

The data that support the findings of this study are available from the corresponding author upon reasonable request.
