# Supplementary material for: Convolutional Neural Network for Fully Automated Cerebellar Volumetry in Children in Comparison to Manual Segmentation and Developmental Trajectory of Cerebellar Volumes
Source: Cerebellum. 2023 Oct 13;23(3):1074–85. doi: 10.1007/s12311-023-01609-2 (PMC11102395; doi:10.1007/s12311-023-01609-2)
Supplement: Supplementary file 1 — (DOCX 204 kb) [file 12311_2023_1609_MOESM1_ESM.docx]

**Supplementary material**


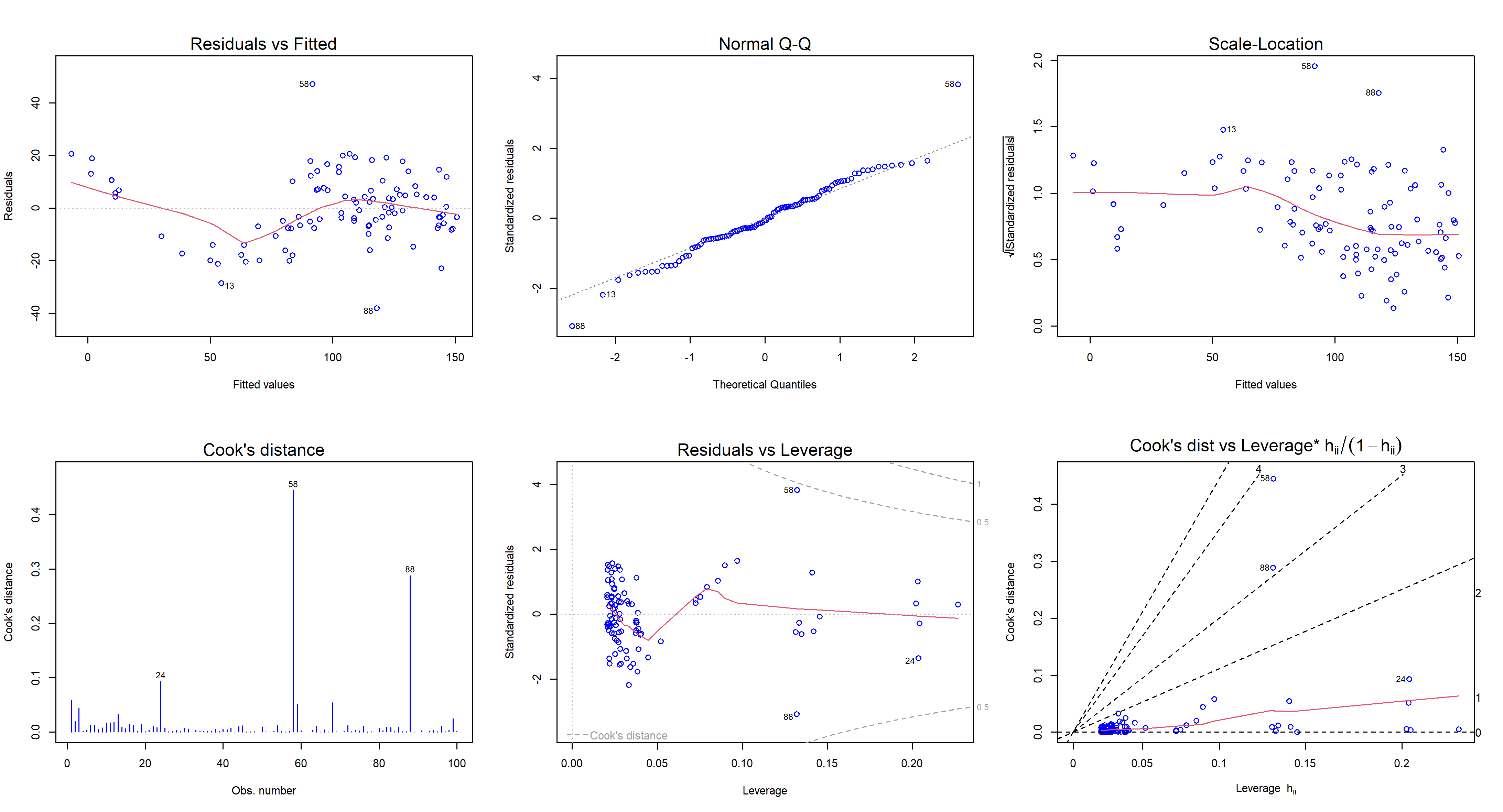
**Regression diagnostic results**

**Figure S1. A series of regression diagnostics for model 1 (Table 3)**

The scatterplot of residuals against fitted values (top left plot) indicates the presence of a non-linear and non-random association for volumes lower than 100 ml which correspond to children younger than 12 months. The Q-Q plot indicates that residuals may be normally distributed, as the points do not deviate severely from the straight dashed line. The normality tests (Shapiro-Wilk and Anderson-Darling) confirmed our observations from the Q-Q plot (p-value > 0.05). According to the scale-location plot (top right plot), there is evidence of heteroscedasticity between 50 and 100 ml since the residuals tend to spread wider and wider around a steep line. The plot on Cook’s distance (bottom left plot) signals three points as outliers that are not influential: Cook’s distance is less than 1 for these points. The scatterplot of standardised residuals versus leverage confirms that there are no influential outliers: all points are found within the bounds illustrated by the grey dashed lines. Only point 58 (female, 34 months old with microcephaly and volume of 138.95 ml) is close to the upper bound of 0.5.
